# Artificial rainfall patterns alter non‐structural carbohydrate allocation to modulate growth and eco‐stoichiometry in *Cyphomandra betacea* seedlings

**DOI:** 10.1111/plb.70152

**Published:** 2025-12-03

**Authors:** X. Li, H. Zeng, L. Sun, H. Guo, X. Cha, Q. Dong

**Affiliations:** ^1^ College of Forestry, Southwest Forestry University Kunming Yunnan China; ^2^ Key Laboratory of National Forestry and Grassland Administration on Biodiversity Conservation in Southwest China, Southwest Forestry University Kunming Yunnan China

**Keywords:** biomass, eco‐stoichiometry, NSC, rainfall pattern

## Abstract

- *Cyphomandra betacea*, a valuable understory crop in southwestern China, exhibits high sensitivity to water availability. Under global climate change with increasingly erratic precipitation, understanding how *Cyphomandra betacea*, seedlings respond to rainfall variations is crucial for sustaining this distinctive industry. Through controlled experiments, this work systematically investigates how different rainfall patterns affect seedling growth and physiology, providing a theoretical basis for science‐based management under future climate scenarios.
- Seedlings were subjected to a four‐month simulated rainfall experiment with two rainfall intervals (T: 3‐day; T_+_: 6‐day) and three rainfall amounts (W: control; W_+_: +40%; W_−_: −40%). Biomass, non‐structural carbohydrates (NSC), and carbon, nitrogen, phosphorus stoichiometric characteristics were analysed.
- Seedling growth is more sensitive to variations in rainfall amount, and appropriate increases in rainfall can promote seedling growth and development. Under changes in rainfall patterns, seedlings prioritize the storage of NSC in stems, followed by leaves, with the lowest allocation to roots. Nitrogen content within organs is pivotal for the composition of NSC and can regulate the sugar‐starch conversion process.
- The July W_+_T treatment resulted in optimal performance for the majority of growth indicators and demonstrated the highest nutrient accumulation efficiency. We identified a stem‐preferential carbon allocation strategy and systemic N limitation, offering key insights for conservation and cultivation under changing climates.

## Graphical abstract

Changes in rainfall patterns severely affect plant growth. *Cyphomandra betacea* seedlings adapt to such changes by adjusting inter‐organ biomass allocation, dynamically regulating stoichiometric characteristics, and non‐structural carbohydrate allocation.

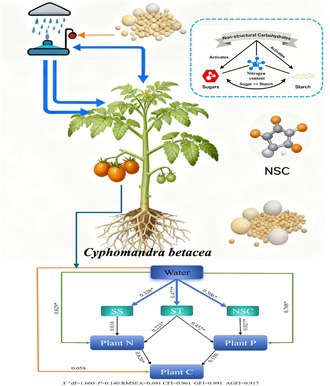

## INTRODUCTION

Global climate warming is significantly altering the spatiotemporal distribution and intensity of rainfall, affecting regional rainfall patterns (both precipitation amount and interval) (Maurer *et al*. 2020; Smith *et al*. 2023). Changes to precipitation regimes alter rainfall frequency, seasonal distribution, and the incidence of extreme precipitation events, directly impacting plant growth and plant adaptive mechanisms to changing rainfall conditions (Deng *et al*. 2021). Understanding how plants will adapt to future climates requires knowledge of the ecological adaptation strategies rooted in internal mechanisms, such as carbon and nutrient balance.

In plant carbon metabolism, non‐structural carbohydrates (NSC), as key products of photosynthesis, serve as an energy source for metabolic processes and contribute to plant structure (Zhao *et al*. 2024). NSC mainly consist of soluble sugars (SS) and starch (ST); their content reflects the plant carbon budget, carbon balance, and stress response capacity. Changes in habitat affect plant competition, reproduction, and survival by regulating the distribution of NSC among organs. Therefore, exploring dynamic changes in NSC is crucial for elucidating mechanisms of storage, transport, allocation, and response to the environment. Previous studies have shown that water stress reduces NSC and soluble sugar content in leaves, stems, and roots of *Populus tomentosa* seedlings (Shangguan *et al*. 2019), and NSC components in leaves of *Quercus variabilis* show significant seasonal fluctuations (Zhang *et al*. 2019). In the absent of precipitation, NSC in leaves of *Larix gmelinii* increases soluble sugars and decreases starch, while the responses in branches lag behind (Du *et al*. 2014). Simultaneously, plant responses to variations in water availability are also reflected in their ecological stoichiometric characteristics. Carbon (C), nitrogen (N), and phosphorus (P) are the main elements in plant ecological stoichiometry, and differences in their concentrations in organs can indicate environmental adaptation strategies for nutrient absorption, allocation, and utilization. Insufficient precipitation often increases plant N:P because the decline in P content exceeds that in N (Wang *et al.* 2019; He & Dijkstra 2014), and inhibition of P uptake is stronger than that of N (Yuan & Chen 2015). Among organs, leaves are particularly sensitive to changes in water availability: insufficient precipitation reduces leaf C:N and increases N:P in both leaves and roots (Sardans *et al*. 2021). These differences are mainly attributed to the magnitude and duration of precipitation and differences in climate (e.g., mean annual temperature and altitude) (Canarini *et al*. 2017; Ren *et al*. 2017; Wilcox *et al*. 2017). However, the interactive effects of rainfall interval and precipitation on carbon–nutrient coordination in economic tree species is poorly understood.

Employing controlled precipitation experiments on ecologically and economically important tree species represents a key approach to overcoming identified knowledge gaps. The tree tomato (*Cyphomandra betacea*; Solanaceae), a perennial evergreen shrub, is native to South America and cultivated in southern China, including Yunnan and southern Tibet. Its fruits are highly nutritious, containing high levels of vitamin C and minerals (Xu *et al*. 2022). Current research on *C. betacea* has primarily focused on seed treatment, fruit composition analysis, fertilization effects, and shade management (Ma *et al*. 2018; Dong *et al*. 2022; Sun *et al*. 2023). However, in its main production area of Yunnan, altered rainfall patterns – uneven rainfall distribution between dry and wet seasons and increasing intermittent droughts – have become important issues, significantly constraining growth and development. To date, little is known about the physio‐ecological mechanisms underlying the response of *C. betacea* to variations in precipitation amount and timing. Most studies have only examined the effects of changes in precipitation amount (e.g., drought stress) or seasonal observations under natural conditions. There remains a considerable lack of understanding regarding how precipitation interval – the frequency and regularity of drought events—will influence plants, and on interactions with precipitation amount. Therefore, this study extends relevant questions from natural ecosystems to agroforestry systems, aiming to fill the research gap on adaptation mechanisms of a specific economic tree species. The objectives were to determine: (1) effects of altered rainfall patterns on growth of *C. betacea* seedlings; (2) responses of NSC, C, N and P concentrations in different organs, and their ecological implications; and (3) adaptation strategies of seedlings in response to reduced rainfall and extended dry intervals. The findings will provide direct guidance for the sustainable cultivation of *C. betacea* by informing irrigation scheduling and nutrient management, thereby assisting farmers in coping with increasingly variable rainfall patterns, and supporting economic stability in production regions.

## MATERIAL AND METHODS

### Plant material and treatments

The material used in this study was 1‐year‐old *C. betacea* seedlings. At the start of the experiment, the average seedling height was 15 cm, with a basal diameter of 0.7 cm. A voucher specimen (IMDY0034602) has been deposited in the Herbarium of Yunnan Branch, Institute of Medicinal Plants, Chinese Academy of Medical Sciences (https://www.cvh.ac.cn/spms/detail.php?id=fbea0cec). The experiment was conducted from April 2023 to July 2023 in the greenhouse at the Southwest Forestry University (Kunming, Yunnan, E102°45′, N25°03′, 1904 m a.s.l.). The greenhouse average annual temperature was 16°C–30°C, relative humidity 23%–67%, atmospheric CO₂ concentration 400–412 ppm, with sufficient light.

In March 2023, *C. betacea* seedlings were transplanted into pots (upper diameter 18 cm, lower diameter 14 cm, height 20 cm). The test soil was a 1:1 mixture of laterite and humus. Each pot was filled with soil to 4/5 of its volume, with ca. 2 kg per pot. The soil had a field capacity of 28.43%, pH 5.62, and contained organic carbon 32.23 g kg^−1^, total N 0.39 g kg^−1^, total P 0.31 g kg^−1^, total K 11.8 g kg^−1^, hydrolysable N 44 mg kg^−1^, available P 11 mg kg^−1^, and available K 61 mg kg^−1^.

### Experimental design

A two‐factorial design yielded six precipitation treatments. Each treatment comprised 18 seedlings, with three replicates, totaling 324 seedlings. The simulated precipitation period spanned April to July, coinciding with the peak growing season of *C. betacea*. Based on meteorological data from Kunming (Lin *et al*. 2024), average precipitation for April–July was set as control (W). Correspondingly, treatments were: 40% increase (W_+_), 40% reduction (W_−_), natural 3‐day precipitation interval (T), and extended 6‐day interval (T_+_). Precipitation volumes per application event were calculated using equation (1). Total precipitation gradients and precipitation interval gradients were simulated by controlling watering frequency and volume per event (Table 1).

(1) $$\text{Precipitation}\;\left(\mathrm{mL}\right)=\begin{array}{l} \mathrm{Pot}\;\text{surface area}\;\left(m^{2}\right)\\ \times \text{Monthly mean rainfall}\;\left(\mathrm{mm}\right)\times 1000 \end{array}$$

**Table 1:** Simulated rainfall and rainfall interval settings.

month	monthly average rainfall (mm)	monthly watering frequency	precipitation interval (day)	precipitation amount (mL)		
				W_−_	W	W_+_
April	25.41	6	T	48	80	112
		3	T_+_	96	160	224
May	20.34	6	T	38	64	90
		3	T_+_	76	128	180
June	146.76	6	T	277	461	645
		3	T_+_	554	922	1290
July	150.06	6	T	283	471	659
		3	T_+_	566	942	1318

T, rainfall duration of 3 days; T_+_, rainfall interval of 6 days; W, average monthly rainfall; W_−_, 40% rainfall reduction; W_+_, 40% water addition.

### Sampling and measurements

#### Plant morphological indicators

A measuring tape was used to measure height of the *C. betacea* seedlings, and a vernier calliper was used to measure diameter at the base of the main stem. The seedlings were separated into roots, stems, and leaves and fresh weights determined using an electronic balance (accuracy 0.01 g) and recorded as root biomass, stem biomass, and leaf biomass. Total biomass was calculated as the sum of these three components.

#### Physiological indicators

For each treatment, three representative samples were selected for destructive sampling. The leaves, stems, and roots were separated and bagged. The samples were enzyme‐inactivated at 105°C then oven‐dried at 80°C to constant weight. Subsequently, samples were ground, sieved, and stored prior to analysis. Total carbon (C) was determined with the potassium dichromate oxidation method. Total nitrogen (N) was determined spectrophotometrically using Nessler's colorimetric method (420 nm). Total phosphorus (P) was determined spectrophotometrically using the vanadium‐molybdenum yellow colorimetric method (450 nm). Soluble sugars were stepwise extracted with 80% ethanol and determined using the phenol‐sulfuric acid method with colorimetric measurement; starch was detected using the same method after enzymatic hydrolysis of residual starch into glucose. The NSC content was the sum of soluble sugar and starch.

### Data analysis

Excel 2021 and SPSS v. 19.0 (SPSS, Chicago, IL, USA) were used for data processing, and graphs were plotted using Origin 2022 (OriginLab, Northampton, MA, USA). Structural equation modelling (SEM) was performed using Amos v. 23.0 to assess the influence of rainfall patterns on seedling growth and physiological parameters. When analysing the relationship between allometric growth, the power function was converted to log y = logβ + αlogx, where α is allotrophic growth index (slope of the equation), β is regression constant, and x and y are biomass of roots, stems, and leaves, respectively, of *C. betacea* seedlings. The slope of the equation was obtained using Standardized Major Axis Estimation (SMA) (Warton and Weber, 2002), which was calculated using SMATR v. 2.0 (Falster *et al.* 2006). Comparing the slope of the equation with the theoretical value of 1.0, allowed estimation of any significant differences between the slope of the SMA and the theoretical value of 1.0. If there were significant differences (*P*_*_1.0*_ < 0.05), this indicates that the allometric growth relationship between biomass of different organs is allometric growth. If this is not significant (*P*_*_1.0*_ > 0.05), it is isometric growth.

## RESULTS

### Growth and biomass

**Fig. 1:**
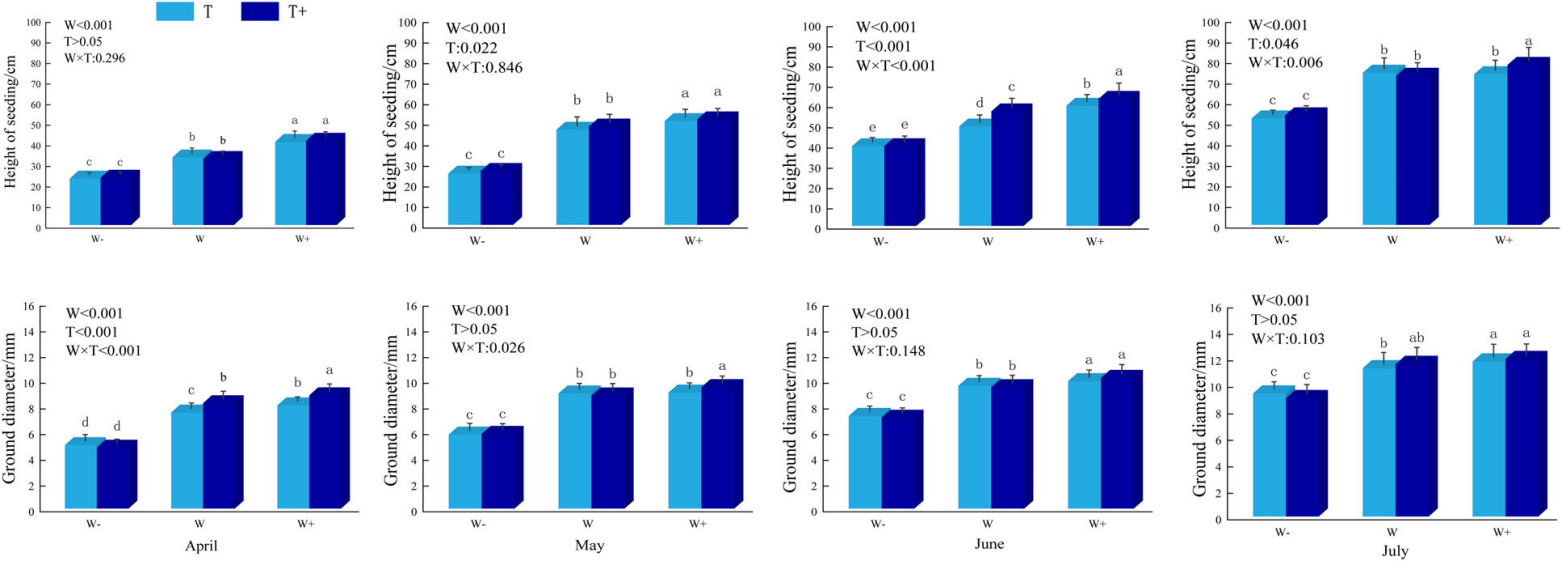
The effect of different rainfall patterns on height and basal diameter of *C. betacea* seedlings. W_−_ (40% decrease in rainfall); W (control); W_+_ (40% increase in rainfall); T (3 day); T_+_ (6 day). Different lowercase letters indicate significant differences between different treatments.

Across all rainfall patterns, both basal diameter and seedling height of *C. betacea* increased significantly with increasing rainfall amount (Fig. 1). Maximum values for these parameters (77.82 cm and 11.89 cm, respectively) were recorded in July under the W_+_T_+_ treatment, indicating that a moderate rainfall increment promoted growth. Statistical analysis confirmed that reduced (W_−_) and increased (W_+_) rainfall significantly inhibited or enhanced, respectively, seedling dimensions (*P* < 0.05), compared to the control (W). In contrast, extending the rainfall interval from T (3 day) to T_+_ (6 day) had no significant effect on either growth metric.

**Fig. 2:**
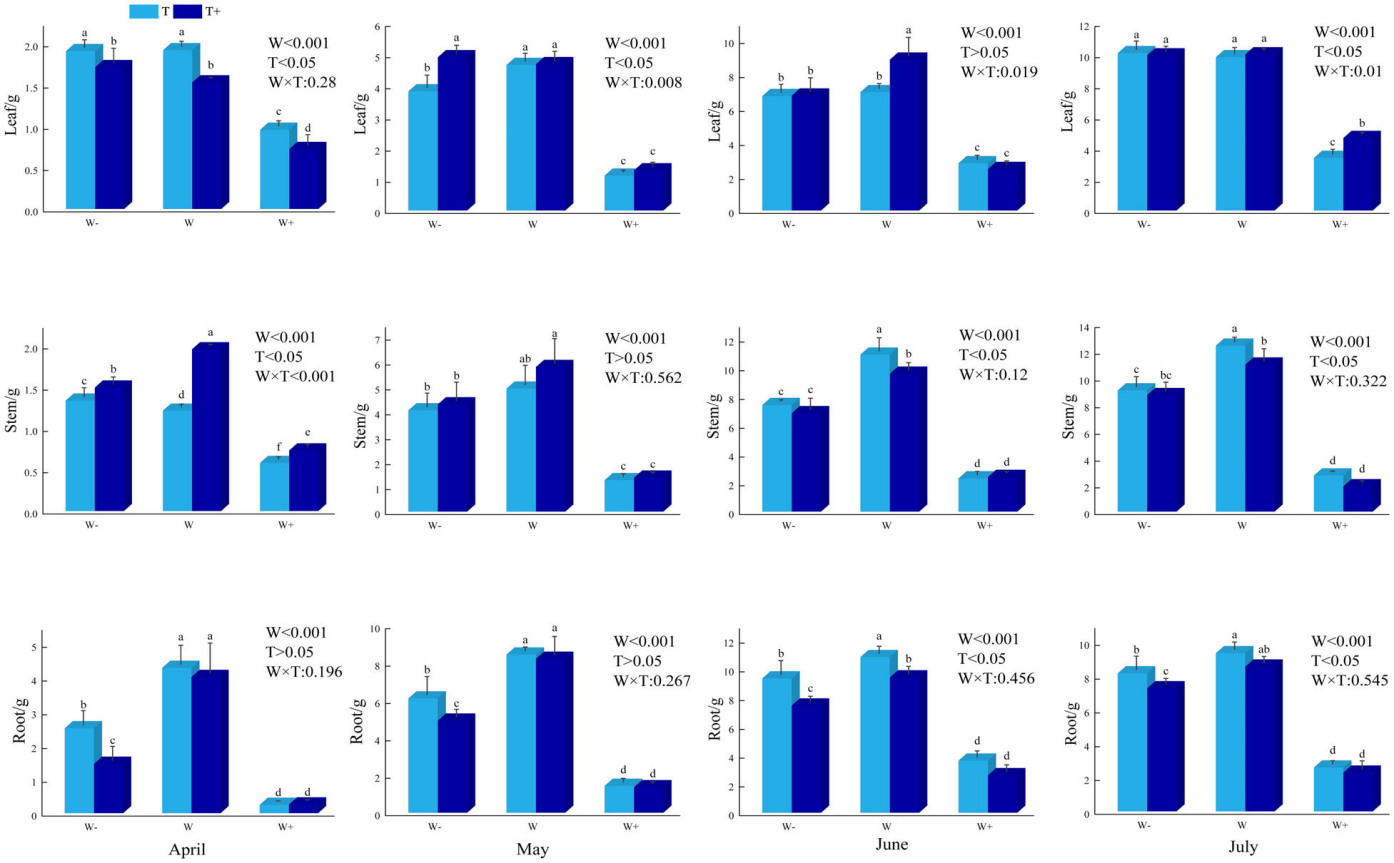
The effect of different rainfall patterns on biomass of various organs in *C. betacea* seedlings.

**Fig. 3:**
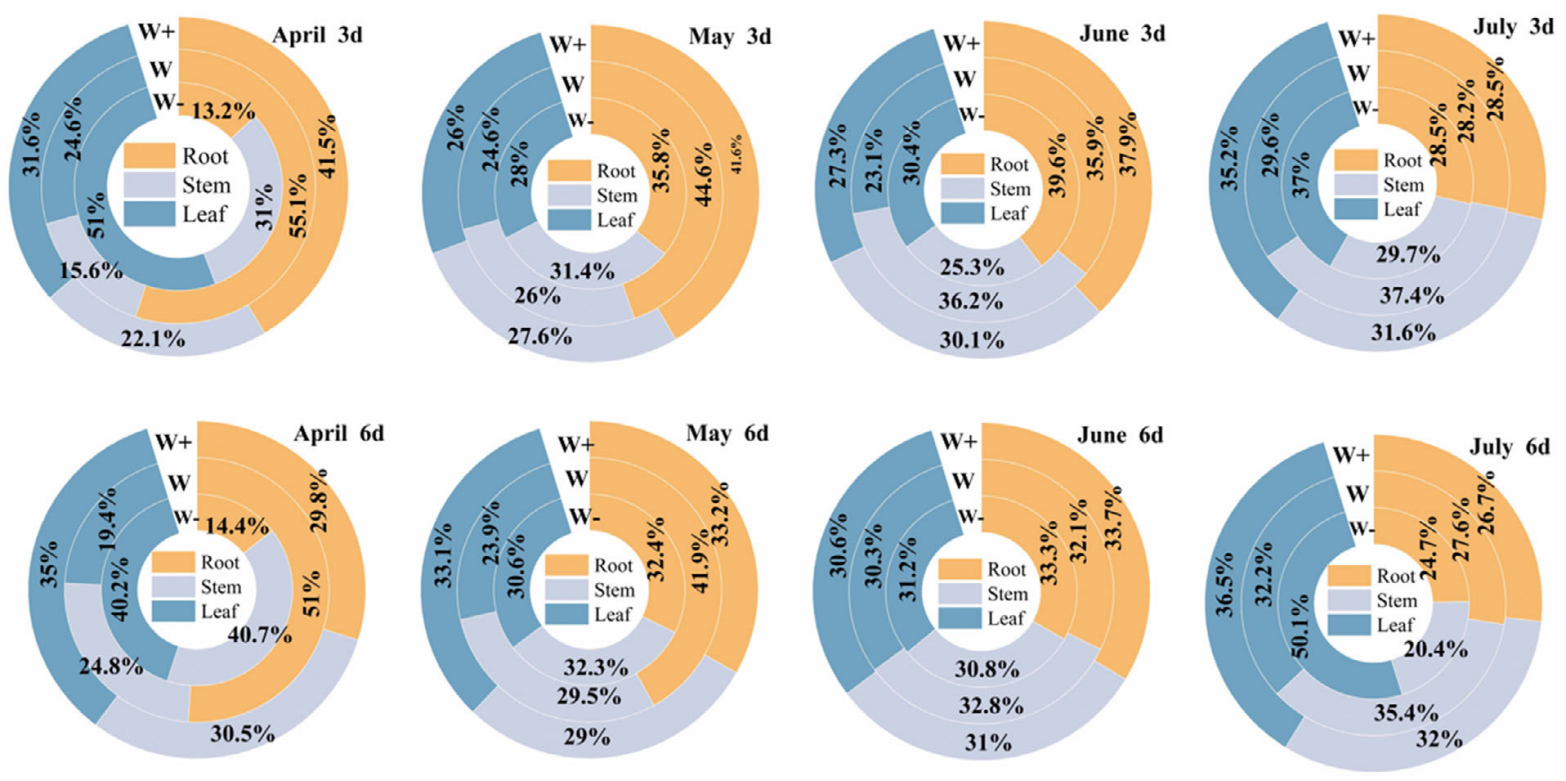
The proportion of biomass in various organs of *C. betacea* seedlings under different rainfall patterns.

Biomass response varied, depending on organ and treatment. Root biomass increased during the first 3 months but decreased significantly in July (*P* < 0.05). In contrast, leaf and stem biomass continued to accumulate and peaked in July. When rainfall amount remained unchanged, extending the rainfall interval led to a reduction in root and stem biomass (Fig. 2). Across all treatments, biomass distribution followed the pattern of root > leaf > stem (Fig. 3), indicating that the plants adopted a survival strategy, prioritizing investment in belowground parts.

### Non‐structural carbohydrates

The content of soluble sugars, starch, and NSC in various organs significantly increased from late May to early June (*P* < 0.05) (Data S1). With the increase in rainfall, the soluble sugar content in various organs generally first rose and then fell and was always significantly higher under the WT treatment compared to other treatments (*P* < 0.05). Starch content in stems and roots peaked under the W_+_T_+_ treatment during April–May, but changed to being highest under the WT treatment during June–July. Results indicated that concentrations of soluble sugars and starch were significantly affected by the interaction of rainfall amount and interval (*P* < 0.05), and under different rainfall patterns, soluble sugar content was consistently highest in the stems and lowest in the roots (*P* < 0.05).

Except for a non‐significant (*P > 0.05*) effect on soluble sugar:starch in leaves in April and July, there were significant (*P <* 0.05) differences in soluble sugar, starch, NSC, and soluble sugar:starch in all organs exposed to different rainfall intervals. Different rainfall intervals had significant (*P* < 0.05) effects on soluble sugar content in roots at different times, and in April and May there were highly significant (*P* < 0.001) effects of the interaction between soluble sugar, starch, NSC, and between soluble sugar:starch on roots throughout the whole experiment (April–July). NSC and soluble sugar:starch were highly significant (*P* < 0.001), and throughout the experiment (April–July), the interaction between these two had a significant (*P* < 0.05) effect on starch in roots, with a gradually decreasing effect on soluble sugar content in stems and leaves (Data S2).

### Stoichiometric characteristics

#### C, N and P content of various organs

**Fig. 4:**
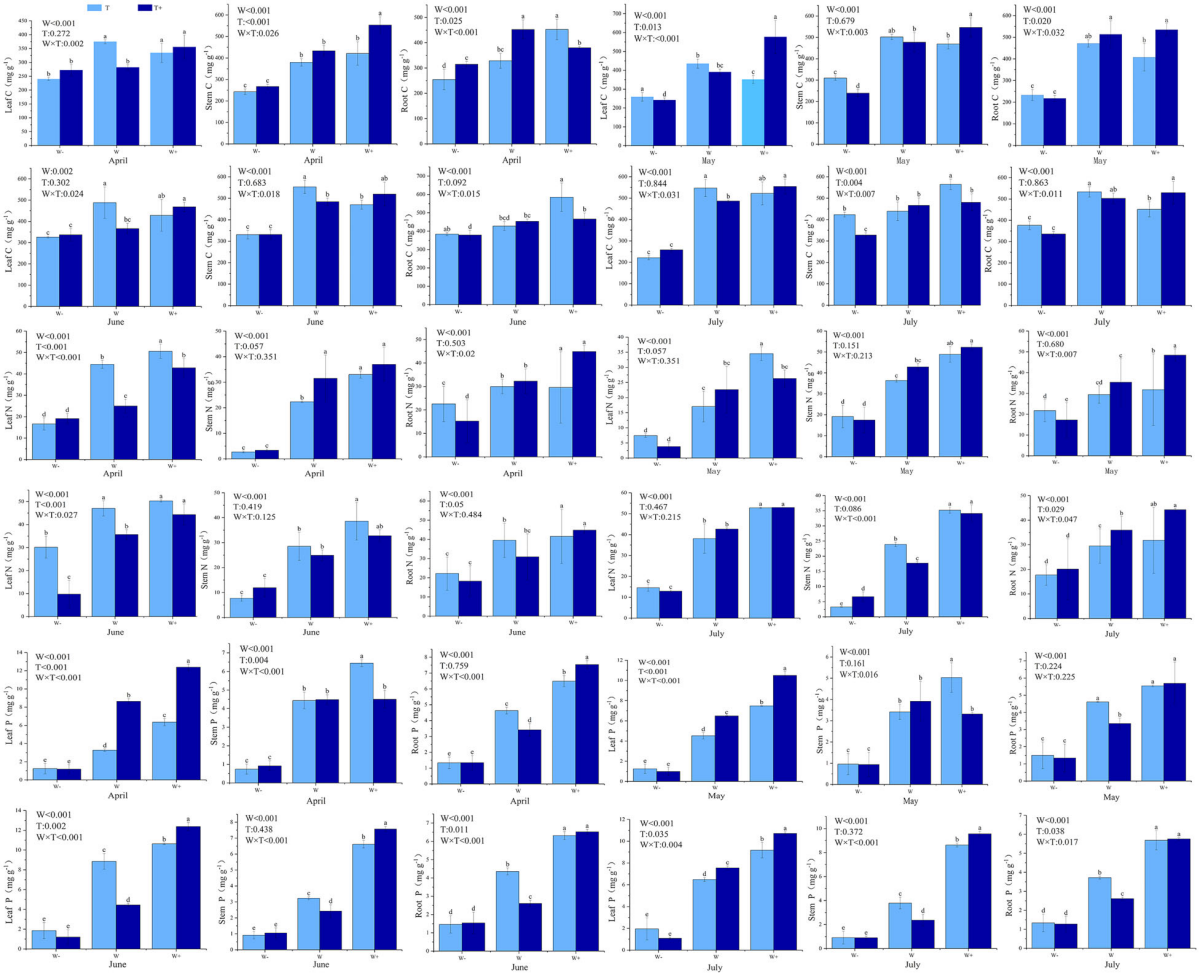
The effect of different rainfall patterns on the content of C, N and P in various organs of *C. betacea* seedlings.

The C, N and P contents in leaves, roots, and stems of *C. betacea* seedlings generally increased with increasing rainfall, although responses were organ‐specific (Fig. 4). Leaf C content exhibited a unimodal trend under the 3‐day intervals (T), peaking in W treatment, with a significant increase from W_−_ to W_+_ (*P* < 0.05). In contrast, at 6 days intervals (T_+_), leaf C rose consistently across the 4 months, reaching a maximum under W_+_. Both N and P content increased with rainfall across all organs and interval treatments, demonstrating consistent nutrient accumulation in response to water availability.

#### Stoichiometric ratios in various organs

**Fig. 5:**
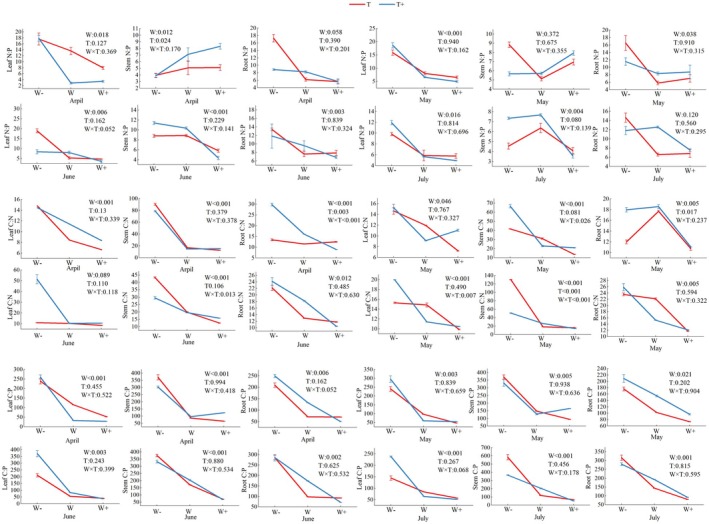
Effect of different rainfall patterns on stoichiometric ratios of various organs of *C. betacea* seedlings.

Under both rainfall intervals, the C:N ratio of organs generally decreased with increasing rainfall, although there were distinct response patterns among different organs (Fig. 5). The leaf C:N increased significantly under extended intervals (T_+_), peaking in June under the W_−_T_+_ treatment. The stem C:N did not show a clear trend, while the root C:N ratio remained relatively stable throughout the experimental period. A similar pattern was observed for C:P ratios: leaf C:P increased significantly under T_+_, reaching a peak in June (W_−_T_+_), whereas stem C:P only responded to extended intervals in July. The root C:P remained stable across all treatments. In contrast, N:P ratios were more variable: both leaf and stem N:P ratios increased under T_+_ conditions, and the timing of peak values varied each month among organs.

#### C, N and P distribution among organs

Under varying rainfall patterns, C, N and P distribution in organs differed (Data S3). Overall, C was highest in roots, followed by leaves and stems, while N and P were highest in leaves, then roots and stems. There were organ‐specific responses: leaf and stem C decreased initially with more rainfall in April before stabilizing, whereas root C declined overall, peaking at 58.18% under W_−_T in April and falling to 26.14% by June. Leaf N and C showed similar trends, reaching 71.91% under W_−_T_+_ in April. Root N and C were higher in April–May but decreased as rainfall increased. In contrast, stem N rose sharply in June, from 28.86% in May to 42.88%. P content trends mirrored C, with root P declining under increased rainfall, being highest at 62.94% in WT (April) and lowest at 18.08% in W_+_T_+_ (July).

#### Allometric growth relationships

Allometric relationships between organ biomass and seedling height–basal diameter in *C. betacea* seedlings were significantly correlated (*P* < 0.01) across precipitation intervals (Table 2). Throughout the experimental period, height–diameter scaling was isometric only under April T treatment, while exhibiting allometric growth (slope >1.0) in other cases. In April, stem–root and leaf–root pairs showed allometric growth, whereas leaf–stem scaling was isometric, indicating reduced rainfall promoted belowground biomass allocation. During May–June, isometric leaf–root scaling suggested enhanced leaf biomass accumulation with increased precipitation. In June, leaf–root and leaf–stem relationships were allometric under T treatment. By July, significantly allometric growth (*P* < 0.05) was observed in all pairs except leaf–root under T treatment.

**Table 2:** Analysis of allometric growth of biomass in organs of *C. betacea* seedlings under different rainfall patterns.

month	trait	interval	allometric index					isometric test		
			*R* ^2^	*P*	slope	95% confidence interval		*F* value	*P*_−0.1_	type
April	Stem(y)‐Root(x)	T	0.892	0.000	0.302a	0.226	0.404	146.311	0.000	*A*
		T+	0.962	0.000	0.364a	0.306	0.433	259.343	0.000	*A*
	Leaf(y)‐Root(x)	T	0.939	0.000	0.266a	0.214	0.332	348.027	0.000	*A*
		T+	0.678	0.006	0.349a	0.214	0.569	34.353	0.001	*A*
	Leaf(y)‐Stem(x)	T	0.948	0.000	0.881a	0.720	1.079	2.168	0.184	*I*
		T+	0.812	0.001	0.959a	0.657	1.400	0.064	0.807	*I*
	Height(y)‐Diameter(x)	T	0.793	0.000	1.091a	0.980	1.216	2.640	0.109	*I*
		T+	0.799	0.000	0.849a	0.763	0.945	9.427	0.003	*A*
May	Stem(y)‐Root(x)	T	0.927	0.000	0.783a	0.616	0.996	5.807	0.047	*A*
		T+	0.956	0.000	0.833a	0.691	1.004	5.360	0.054	*I*
	Leaf(y)‐Root(x)	T	0.962	0.000	1.038a	0.874	1.234	0.264	0.623	*I*
		T+	0.896	0.000	0.990a	0.745	1.316	0.007	0.938	*I*
	Leaf(y)‐Stem(x)	T	0.962	0.000	1.038a	0.874	1.234	0.264	0.623	*I*
		T+	0.896	0.000	0.990a	0.745	1.316	0.007	0.938	*I*
	Height(y)‐Diameter(x)	T	0.860	0.000	1.438a	1.316	1.570	69.901	0.000	*A*
		T+	0.868	0.000	1.363a	1.250	1.486	52.496	0.000	*A*
June	Stem(y)‐Root(x)	T	0.968	0.000	1.347a	1.149	1.580	20.003	0.003	*A*
		T+	0.972	0.000	1.034a	0.891	1.200	0.276	0.616	*I*
	Leaf(y)‐Root(x)	T	0.969	0.000	0.872a	0.746	1.022	4.159	0.081	*I*
		T+	0.923	0.000	0.985a	0.771	1.260	0.018	0.896	*I*
	Leaf(y)‐Stem(x)	T	0.938	0.000	0.647a	0.520	0.808	22.643	0.002	*A*
		T+	0.949	0.000	0.953a	0.781	1.165	0.309	0.595	*I*
	Height(y)‐Diameter(x)	T	0.726	0.000	1.166a	1.031	1.319	6.168	0.015	*A*
		T+	0.787	0.000	1.268a	1.136	1.415	18.924	0.000	*A*
July	Stem(y)‐Root(x)	T	0.985	0.000	1.138a	1.021	1.268	7.976	0.026	*A*
		T+	0.957	0.000	1.301a	1.081	1.565	11.383	0.012	*A*
	Leaf(y)‐Root(x)	T	0.960	0.000	0.889a	0.745	1.062	2.416	0.164	*I*
		T+	0.969	0.000	0.599a	0.513	0.701	64.694	0.000	*A*
	Leaf(y)‐Stem(x)	T	0.937	0.000	0.781a	0.626	0.977	6.825	0.035	*A*
		T+	0.973	0.000	0.461a	0.399	0.533	192.427	0.000	*A*
	Height(y)‐Diameter(x)	T	0.505	0.000	1.425a	1.207	1.682	18.540	0.000	*A*
		T+	0.629	0.000	1.174a	1.016	1.357	4.897	0.030	*A*

*P*_−1.0_ represents a significant difference between slope and theoretical value of 1.0. Different letters indicate significant differences in biomass of various organs at different rainfall durations (*P* < 0.05). A: allometric relationship, *I*: isometric relationship. *R* ^2^ coefficient of determination, *P* significance.

#### Structural equation model

**Fig. 6:**
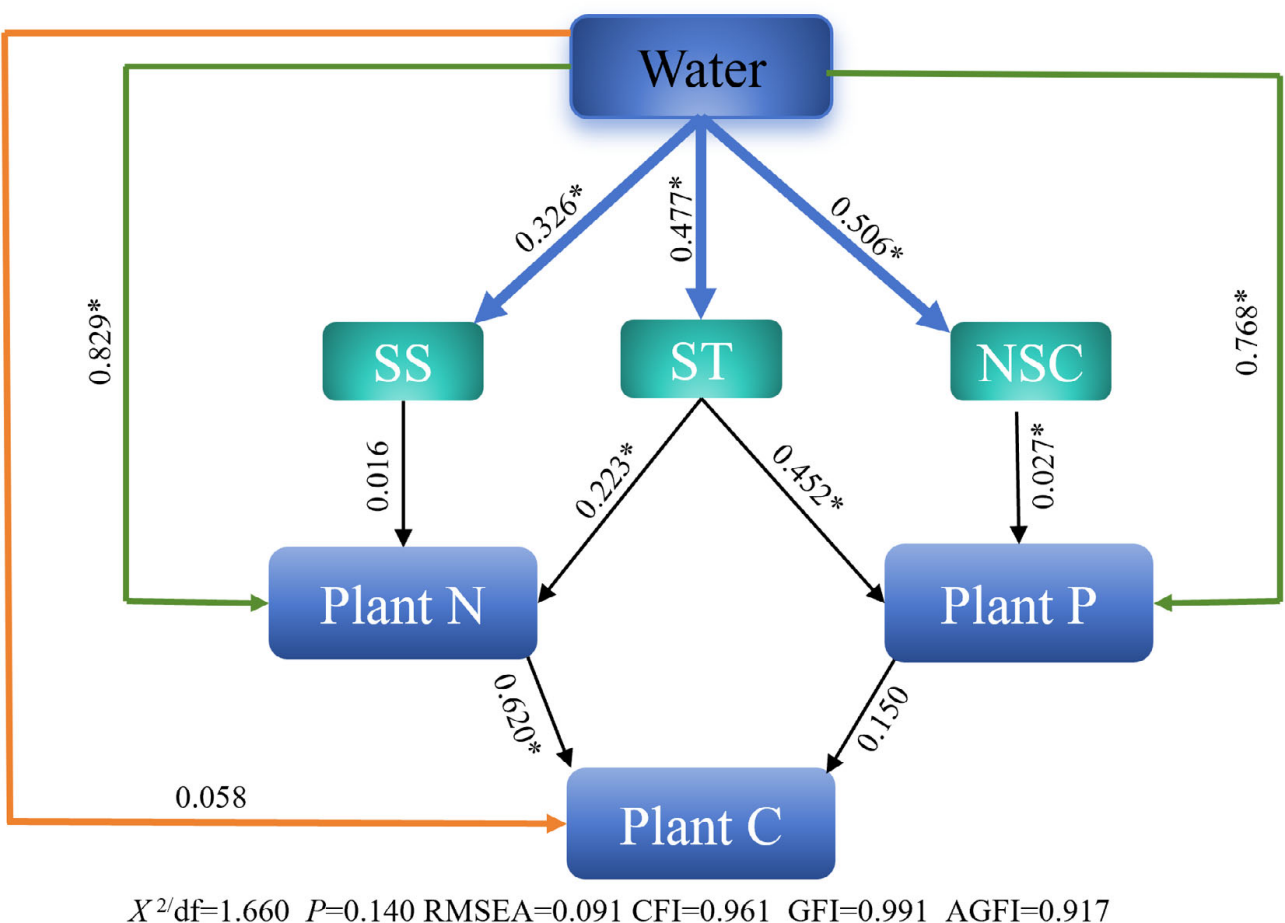
Structural equation modelling (SEM) of different rainfall patterns on various indicators of *C. betacea* seedlings.

The structural equation modelling (SEM) indices showed a good model fit (*X* ^2^/df = 1.660, RMSEA = 0.091, CFI = 0.961), indicating that the model adequately represents causal relationships among the variables (Fig. 6). Variations in precipitation had significant positive effects (*P* < 0.05) on NSC, plant N content, and plant P content of *C. betacea*, with path coefficients of 0.506, 0.829, and 0.768, respectively. However, the effect on plant C content was not significant (path coefficient = 0.058, *P* > 0.05). Further analysis revealed that starch had a significant positive effect on both Plant N and Plant P (path coefficients = 0.223 and 0.452, *P* < 0.05). Concurrently, Plant N had a significant positive effect on Plant C (path coefficient = 0.620, *P* < 0.05). In contrast, the effect of soluble sugars on Plant N was not significant (path coefficient = 0.016, *P* > 0.05). These results indicate that increased rainfall directly facilitates accumulation of NSC, N and P in plants. Within this process, starch positively drives accumulation of N and P, while N content further promotes accumulation of C.

### Correlation analysis

**Fig. 7:**
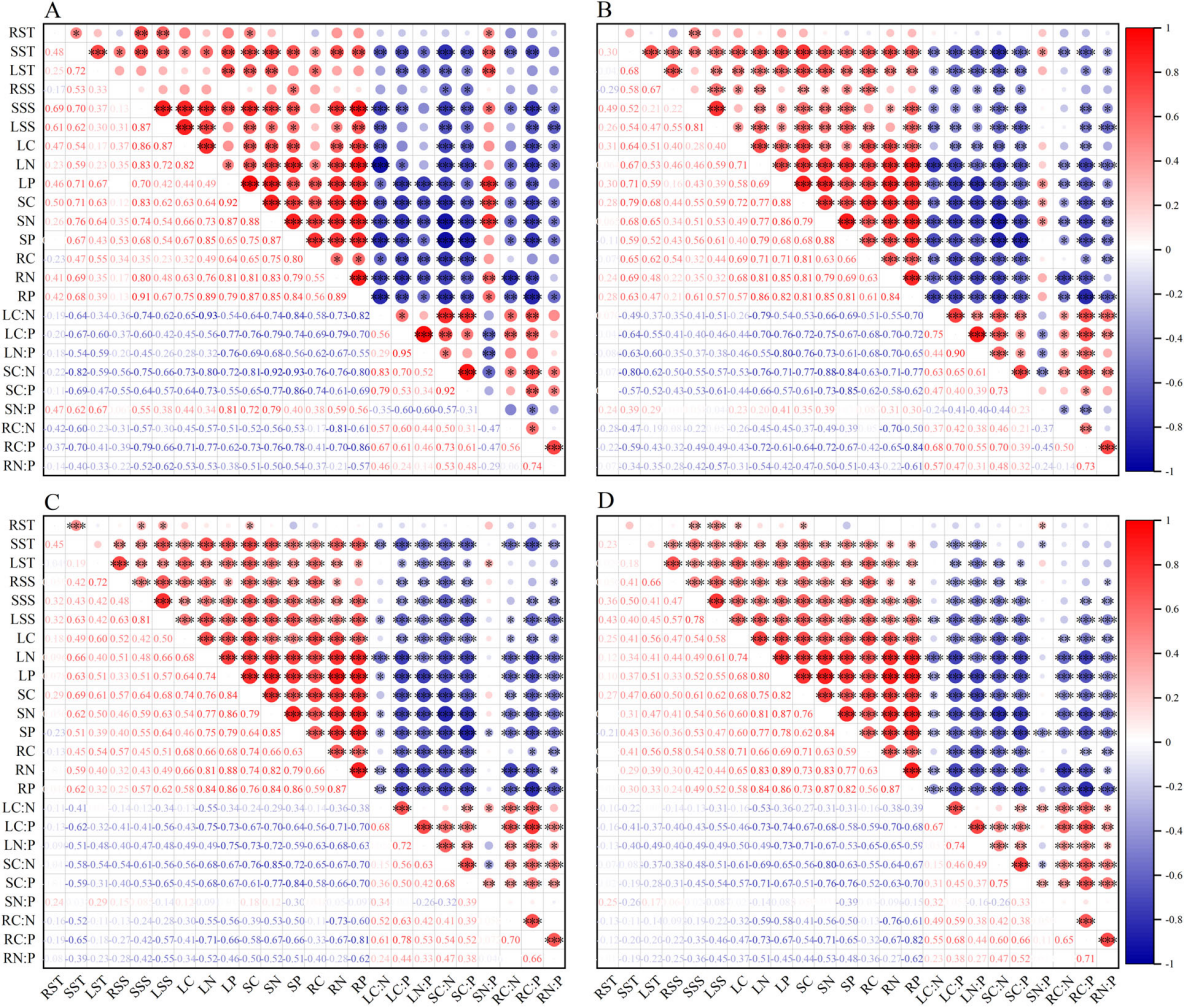
Correlation analysis between NSC composition and C, N, P and their stoichiometric ratios of *C. betacea* seedlings under different rainfall treatments. A, B, C and D: April, May, June and July. RST: root starch content. SST: stem starch content. LST: leaf starch content. RST: root soluble sugar content. SSS: stem soluble sugar content. LSS: leaf soluble sugar content. LC: leaf carbon content. LN: leaf nitrogen content. LP: leaf phosphorus content. SC: stem carbon content. SN: stem nitrogen content. SP: stem phosphorus content. RC: root carbon content. RN: root nitrogen content. RP: root phosphorus content. LC:N: carbon‐to‐nitrogen ratio in leaves. LC:P: carbon‐to‐phosphorus ratio in leaves. LN:P: nitrogen‐to‐phosphorus ratio in leaves. SC:N: carbon‐to‐nitrogen ratio in stems. SC:P: represents the carbon‐to‐phosphorus ratio in stems. SN:P: nitrogen‐to‐phosphorus ratio in stems. RC:N: carbon‐to‐nitrogen ratio in roots. RC:P: carbon‐to‐phosphorus ratio in roots. RN:P: nitrogen‐to‐phosphorus ratio in roots.

Pearson correlation revealed dynamic relationships among nutrients and carbohydrates in *C. betacea* seedlings (Fig. 7). In April, stem N:P was significantly positively correlated with C, N, and P across all organs. By May, it was positively correlated specifically with stem C and N content. In July, this relationship reversed, with stem N:P becoming negatively correlated with stem P and C. Additionally, leaf N was consistently negatively correlated with stoichiometric ratios (stem N:P, C:P and N:P) in leaves.

## DISCUSSION

### Plant growth and biomass

This study first evaluated the effects of rainfall patterns on seedling growth. As outlined above, elucidating the interaction between rainfall interval and rainfall amount was the core objective of this research, with growth response serving as primary indicator of its biological effects. Plant basal diameter, height and biomass are direct indicators of growth (Chen *et al*. 2024). Studies have shown that increased precipitation improves soil moisture and promotes plant height and basal diameter growth of *Stipa grandis* seedlings (Zhou *et al*. 2010). In the present study, seedling height and basal diameter of *C. betacea* seedlings increased with increasing precipitation, which is consistent with previous research.

Numerous studies have shown that increasing rainfall generally enhances soil moisture content and effectively increases plant productivity (Zhang *et al*. 2021; Zeng *et al*. 2024). Infrequent, but large, rainfall events at long intervals can increase deep soil infiltration and reduce evaporative loss, which is conducive to accumulation of plant biomass (Wilschut & van Kleunen 2021; Li *et al*. 2024b). In this study, extension of the rainfall interval from T to T_+_ led to deeper water infiltration, resulting in reduced root biomass of *C. betacea*. This is consistent with findings for altered rainfall timing in the relatively humid prairies of North America (Fay *et al*. 2000), but contrary to the results of Zhou *et al*. (2010) for effects of rainfall changes on *Stipa grandis* seedlings in typical grasslands of Inner Mongolia. This could be because underdeveloped root systems are unable to utilize deep soil water, leading to decreased water use efficiency. Therefore, shortening the rainfall interval can effectively promote *C. betacea* root growth in the short term.

Stem biomass increased significantly with prolonged precipitation intervals during the dry season (April–May), whereas the opposite trend was observed during the rainy season (June–July). This may be because excess rainfall in the rainy season can lead to overly wet soil, displacement of soil air, leading to impaired root water uptake. Additionally, intense rainfall can cause loss of both water and nutrients, preventing plants from adequately absorbing nutrients and water (Yuan *et al*. 2019).

Leaf biomass was not significantly affected by rainfall intervals, suggesting that nonlinear response mechanisms require further investigation. When rainfall increased from W‐ to W, the biomass of all organs in *C. betacea* seedlings increased, which is consistent with most studies (McDowell 2011); however, when rainfall increased to W+ (+40%), biomass significantly decreased, mainly because excess water inhibits cell elongation, reduces leaf area, and lowers net photosynthesis rate, ultimately slowing growth (Yuan *et al*. 2022).

### Non‐structural carbohydrates

As a key product of photosynthesis, NSC regulate metabolism, growth, and stress resistance (Blumstein *et al*. 2022). Plants have survival strategies by regulating NSC and its component parts in different organs to respond to environmental stresses such as rainfall variation, thereby affecting physiological characteristics and growth mechanisms (Hesse *et al*. 2021). In this study, changes in rainfall significantly affected the NSC content in different organs of *C. betacea* seedlings (*P* < 0.05), while rainfall interval had no significant effect. At fixed intervals, increasing the amount of rainfall produced a unimodal response in NSC content: initially promoting the accumulation of photosynthetic C, but excess rainfall then inhibiting metabolic translocation. As seasonal rainfall increased, the NSC allocation pattern was stem > leaf > root, reflecting the preferential supply of C resources to water transport and nutrient allocation in stems, followed by leaves, with the lowest C input to roots due to the absorption function requirements. This strategy helps plants better adapt to and cope with water abundance or even excess.

As the main storage substance in plants, starch can be converted into soluble sugars and transported between organs under stress, ensuring normal physiological activity (Hajihashemi *et al*. 2020). Under varying rainfall, changes in starch in different organs of *C. betacea* seedlings differed from those of soluble sugars. With increased precipitation, the soluble sugar content in all organs except leaves increased: both leaf and stem starch increased, while root starch remained unchanged. This may be because, as rainfall increases, plants allocate more photosynthetic products towards the synthesis of starch. The increased starch content in stems supports their structural stability and strength, while the primary function of roots is to absorb water and nutrients, resulting in lower and relatively stable soluble sugar and starch content in roots. This indicates that, throughout the changes in rainfall, the stem serves as the main non‐structural C storage organ. The soluble sugar:starch ratio is an indicator of the efficiency of transformation between soluble sugars and starch: a higher value indicates greater demand for soluble sugars in the plant (Van Brenk *et al*. 2024). This study found that under different rainfall treatments, soluble sugar:starch values in various organs of *C. betacea* ranged from 1.14 to 17.91, all exceeding 1. This suggests that soluble sugar occupies a dominant position in C allocation, reflecting a strategic trade‐off at the organ level towards immediate metabolic demands.

### C, N and P stoichiometry

There are differences in organ allocations in the response of plant nutrients to changes in water availability (Yang *et al*. 2014). In this study, the content of C, N, and P in each organ of *C. betacea* increased with increasing precipitation, while the interval between rainfall events did not have a significant effect. This may be related to immature development of the root system or insufficient nutrient absorption efficiency within a short‐term rainfall interval, resulting in no significant change in element content of organs. The variation in C content was smaller than that of N and P, indicating that C, as an essential element in plant organs, has some level of stability. Previous studies generally found that plants increase leaf N content under drought conditions to enhance cell osmotic pressure, thereby improving water use efficiency (Guo *et al*. 2025b). But in this study, the observed increase in leaf N content of *C. betacea* seedlings with increased precipitation contradicts this, most likely because increased rainfall raised the available N content in the soil, enabling *C. betacea* seedlings to absorb and utilize N more efficiently, thus promoting accumulation of N in the leaves. Phosphorus, as a key component of phospholipids and nucleic acids, is essential for plant growth and development. High P content can increase growth rates and ecological competitiveness (McDowell 2011). In this experiment, P content in all organs of *C. betacea* increased with increased precipitation, confirming the positive effect of P on growth. Leaf P content was significantly higher than that in roots and stems, mainly because as the core organ of photosynthesis, leaves require a large amount of P for energy conversion and physiological metabolism (Wang *et al*. 2023). However, the mechanisms underlying interspecific differences in organ allocation of key elements need further investigation.

Stoichiometric characteristics are significantly related to plant growth, among which C:N and C:P are key indicators for characterizing growth rate and are usually negatively correlated (Fan *et al*. 2015; Zhu *et al*. 2017). In this study, increased precipitation significantly reduced C:N and C:P ratios in organs of *C. betacea*, indicating that seedling growth accelerated with abundant water. Combined with SEM analysis, the path coefficients of rainfall on N and P content (0.829 and 0.768) are significantly higher than its effect on C content (0.058; *P* > 0.05), and N content has a significantly positive effect on C content (0.620; *P* < 0.05), indicating that rainfall mainly promotes the absorption of N and P, indirectly drives C fixation, and ultimately leads to the decrease of C:N and C:P ratios. The leaf N:P ratio serves as a diagnostic indicator of nutrient limitation: N:P 14 indicates P limitation, while values between 14 and 16 suggest co‐limitation by N and P (Koerselman & Meuleman 1996). Both the leaf and stem N:P ratios of *C. betacea* seedlings were below 14, reflecting an overall N limitation. Accordingly, it is suggested that, in commercial production, N fertilizer should be appropriately increased and water deficiency avoided to improve yield. In addition, both leaf and stem N:P ratios of *C. betacea* seedlings were below 14 and positively correlated with NSC content of each organ, indicating that changes in organ N content predominantly regulate NSC and its component content and drive interconversion between soluble sugar and starch. However, with increasing rainfall, this correlation was significantly weakened, confirming that changes in N content can serve as a key indicator for analysing C metabolism and growth status of *C. betacea* seedlings.

### C, N and P partitioning among organs

Experiments have shown that the allocation patterns of C, N, and P in *C. betacea* seedlings are organ‐specific: the C content follows the order root > leaf > stem, while the N and P content follow leaf > root > stem. This pattern is primarily driven by organ functions: roots, as the main absorption organs, require high C content to support growth and synthesis of organic matter (Li *et al*. 2024a); leaves, being the central organs of photosynthesis, preferentially accumulate N to meet metabolic demand and maintain a high N content through efficient uptake and transport of nutrients from the roots (He *et al*. 2007). As for stems, serving as transport and support organs, their C, N and P content remain relatively stable. Notably, root C content decreased with increasing rainfall, possibly related to suppressed respiration and reduced organic matter absorption caused by soil hypoxia (Guo *et al*. 2025a). Leaf P content changed little under different rainfall treatments, reflecting the stable demand for P as a key metabolite. Under different rainfall regimes, the overall nutrient allocation in *C. betacea* seedling organs shows that increased rainfall favours N allocation to stems and P allocation to leaves, while prolonged intervals between rainfall events promote C and N allocation to leaves.

## CONCLUSIONS

This study elucidates the response strategies of *C. betacea* seedlings to changes in rainfall patterns, identifying the simulated July W + T treatment as the optimal approach for promoting seedling growth. Seedling growth is significantly regulated by rainfall amount: a moderate increase in rainfall promoted growth, whereas excess rainfall had an inhibitory effect. Carbon allocation consistently prioritized stems, with soluble sugars being dominant. Growth of *C. betacea* seedlings is constrained by N limitation throughout growth stages, as indicated by leaf N:P values consistently below 14. These conclusions directly inform seedling water management for *C. betacea*, leading to recommendation of a precise ‘water–fertilizer coupling’ strategy, avoiding excess irrigation while increasing N fertilizer application to boost productivity and resource efficiency. Future research requires long‐term field studies, especially on the yield of mature trees. This work establishes a foundation for sustainably cultivating this economic species under climate change.

## AUTHOR CONTRIBUTIONS

XL wrote the manuscript, QD designed the experiments, provided critical revisions and final approval of the article. HZ carried out the experiments and ran the data, HG, XC and LS participated in measurement of the indicators. All authors contributed to the final version of the manuscript.

## FUNDING INFORMATION

This research was funded by Yunnan Province ‘Three Regions’ Science and Technology Talent Support Program (grant no. 20200402) and Fund of Southwest Forestry University (grant no. 2020YY013). We thank the Foundation of Economic Support. The funding organizations were not involved in the design of the study, data collection, analysis of the data, or the writing of the manuscript.

## CONFLICTS OF INTEREST

The authors declare no conflicts of interest.

## Supporting information


**Data S1.** Effects of Different Rainfall Patterns on NSC in Various Organs of *C. betacea* seedlings.

**Data S2.** Two‐factor ANOVA on the effect of different rainfall patterns on the content of non‐structural carbohydrates in various organs of *C. betacea* seedlings (*F*‐value).

**Data S3.** Effect of different rainfall patterns on C, N and P partitioning among organs of *C. betacea* seedlings.

## Data Availability

The data underlying this article will be shared on reasonable request to the corresponding author.
