# Supplementary figures and images for: Artificial rainfall patterns alter non‐structural carbohydrate allocation to modulate growth and eco‐stoichiometry in Cyphomandra betacea seedlings
Source: Plant Biol (Stuttg). 2025 Dec 3;28(2):487–97. doi: 10.1111/plb.70152 (PMC12884024; doi:10.1111/plb.70152)

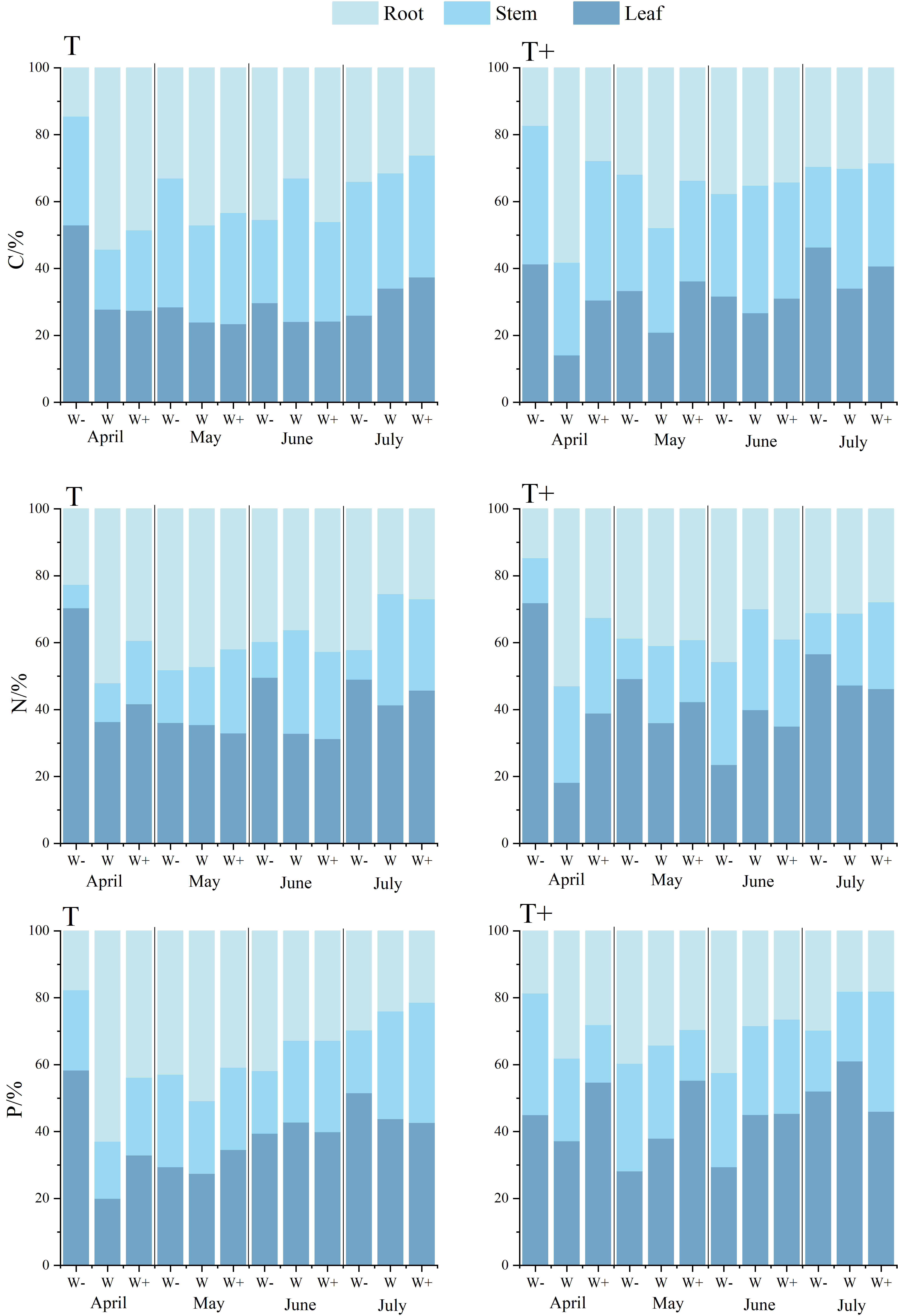

Supplement: Supplementary file 3 — Data S3. Effect of different rainfall patterns on C, N and P partitioning among organs of C. betacea seedlings. [file PLB-28-487-s002.png]
